# Prostate Stromal Cells Express the Progesterone Receptor to Control Cancer Cell Mobility

**DOI:** 10.1371/journal.pone.0092714

**Published:** 2014-03-24

**Authors:** Yue Yu, Jennifer Suehyun Lee, Ning Xie, Estelle Li, Antonio Hurtado-Coll, Ladan Fazli, Michael Cox, Stephen Plymate, Martin Gleave, Xuesen Dong

**Affiliations:** 1 Department of Urologic Sciences, University of British Columbia, Vancouver, British Columbia, Canada; 2 Department of Medicine, University of Washington School of Medicine and VAPSHCS-GRECC, Seattle, Washington, United States of America; 3 Department of Obstetrics and Gynaecology, University of Toronto, Toronto, Ontario, Canada; UC Davis Comprehensive Cancer Center, United States of America

## Abstract

**Background:**

Reciprocal interactions between epithelium and stroma play vital roles for prostate cancer development and progression. Enhanced secretions of cytokines and growth factors by cancer associated fibroblasts in prostate tumors create a favorable microenvironment for cancer cells to grow and metastasize. Our previous work showed that the progesterone receptor (PR) was expressed specifically in prostate stromal fibroblasts and smooth muscle cells. However, the expression levels of PR and its impact to tumor microenvironment in prostate tumors are poorly understood.

**Methods:**

Immunohistochemistry assays are applied to human prostate tissue biopsies. Cell migration, invasion and proliferation assays are performed using human prostate cells. Real-time PCR and ELISA are applied to measure gene expression at molecular levels.

**Results:**

Immunohistochemistry assays showed that PR protein levels were decreased in cancer associated stroma when compared with paired normal prostate stroma. Using *in vitro* prostate stromal cell models, we showed that conditioned media collected from PR positive stromal cells inhibited prostate cancer cell migration and invasion, but had minor suppressive impacts on cancer cell proliferation. PR suppressed the secretion of stromal derived factor-1 (SDF-1) and interlukin-6 (IL-6) by stromal cells independent to PR ligands. Blocking PR expression by siRNA or supplementation of exogenous SDF-1 or IL-6 to conditioned media from PR positive stromal cells counteracted the inhibitory effects of PR to cancer cell migration and invasion.

**Conclusions:**

Decreased expression of the PR in cancer associated stroma may contribute to the elevated SDF-1 and IL-6 levels in prostate tumors and enhance prostate tumor progression.

## Introduction

Prostate tumors have multiple cell populations. Cancer cells are surrounded by non-epithelial cellular environment consisting of fibroblasts, smooth muscle cells and myofibroblasts. Accumulated evidences show that reciprocal epithelium-stroma interactions are critical for tumor development, growth and metastasis [1], [2]. For example, the benign prostatic epithelial cell line BPH-1 is usually nontumorigenic in nude mice. However, when combined with carcinoma associated fibroblasts (CAFs) and grafted into renal capsule, BPH-1 cells formed tumors [3]. These findings demonstrate that stromal cells play crucial roles in malignant transformation. Through secreting cytokines and growth factors, CAFs also provide a supportive microenvironment to facilitate tumor growth, invasion and metastasis [4], [5]. However, despite these critical roles of stroma in prostate cancer (PCa), the therapeutic strategy targeting prostate stroma is greatly under appreciated. This reflects our limited knowledge on stroma-epithelium interactions at the cellular and molecular levels.

It is known that cancer associated stroma enhances secretion of multiple cytokines, which are important components of the tumor microenvironment [6]. Stromal cell derived factor-1 (SDF-1) is secreted by stromal fibroblasts and acts by binding to its receptor, CXCR4, on the membrane of epithelial cells to trigger multiple signal pathways [7]–[10]. The SDF-1/CXCR4 axis has been shown to facilitate cancer cell invasion, tumor angiogenesis [11], [12], stimulate cell proliferation [13], [14] and protect cells from chemotherapeutic drug-induced apoptosis [15]–[17]. SDF-1 mRNA levels are increased in cancer tissues when compared with adjacent benign tissues [18] and are the highest in metastatic PCa [19]. Moreover, CXCR4 expression is also elevated in PCa tissues [19], further amplifying the actions of SDF-1. Interleukin 6 (IL-6) is also an important cytokine that can stimulate the Janus Kinases/Signal Transducer and Activator Transcription 3 pathway in cancer cells [20]. Both SDF-1 and IL-6 can activate the androgen receptor (AR) at low levels of androgens in PCa cells and contribute to tumor progression to the castration resistant stage [21]–[23]. IL-6 was reported to enhance PCa cell proliferation and protect cells from apoptosis in tumor xenografts [24], [25]. Elevated serum IL-6 levels were also shown to be a poor prognosis marker [26], [27].

Prostate stromal cells also express several steroid receptors including the androgen and the estrogen receptors. These receptors were reported to be important for stromal cells to direct PCa development through modulating expression of cytokines/chemokines [28]–[30]. We recently reported that both progesterone receptor (PR) isoforms, PRA and PRB, were expressed specifically in prostate stroma and negatively regulated stromal cell proliferation [31]. In this study, we expanded our efforts to measure PR protein levels in PCa and PR regulation of SDF-1 and IL-6 expression in prostate stromal cells.

## Materials and Methods

### Human Prostate Tissues and Immunohistochemistry

Twenty-seven whole mount sections of human prostate tissue biopsies were obtained from radical prostatectomies. Detailed information on each tissue sample was listed in Table 1. All patients signed an informed consent to a protocol that was reviewed and approved by the UBC Clinical Research Ethics Board (Certificate #: H09-01628). Immunohistochemistry (IHC) assays were performed using Ventana Discovery XT autostainer (Ventana Medical Systems) with antibodies against PR (AbCam) and PRB (Cell Signaling) as reported [31]. Digital images of tissue slides were scanned by a BLISS scanner system (Bacus Lab Inc). Within the peripheral zones, 5 stromal fields (>1000 nuclei) were chosen by pathologist (L.F.) adjacent to benign epithelial cells and 5 other stromal fields were from adjacent cancerous epithelial cells. Five other stromal fields were from the transition zones. The pathological scores were achieved by the software Digital Image Hub (Leica biosystem) to calculate the percentage of stained nuclei and the staining intensity. The relative levels of PR and PRB were calculated as the index of HSCORE = ∑pi(i+1), where i = the intensity of staining, and pi = the percentage of stained cells. HSCOREs were used to compare PR and PRB levels between normal and cancer stroma and between peripheral zones and transition zones.

**Table 1:** Pathological Parameters of Study Patients and PSA Levels.

Distiller ID	Age	Pathological Stage	Biopsy Gleason Overall	Gleason Score	Diagnosis PSA	PSA Recurrence
4030	69	T3A	7	7	15	NO
3939	46	T2C	6	7	3.6	NO
3058	67	T2C	7	7	9.8	NO
1700	88		8			
1566	70	T2C		9		
3335	68	T2C	6	6	8	
1581	64	T3B	8	9	11.5	
3157	64	T2C	7	6	6.9	NO
3335	68	T2C	6	6	8	
2917	65	T2C	6	7	12.1	
2656	59	T3B	9		15.1	YES
2012	60	T2A	7			
1695	62	T3A	6		22	YES
1500	63	T3C	7	9	11	YES
2091	66	T2C	7		1.2	NO
1603	56	T2			4.6	
1500	63	T3C	7	9	11	YES
1665	67	T2	6		31	NO
2619	63	T2C	6	6	2.7	NO
1626	66	T2	8	7	40	NO
1684	63	T2A	9		6.3	YES
2785	64	T2		7		
1185	69	T2C	6	6	22.6	YES
2168	63	T3	9		11.2	NO
2734	57	T3A	7		7.2	YES
2926	46	T2C	7	7	19.7	YES
2878	65	T2C	7	6	1.68	NO

### Prostate Stromal Cell Lines

Human prostatic stromal cell line (WPMY-1) and PCa cell lines LNCaP and PC-3 were purchased from American Type Culture Collection (Manassas, VA). LNCaP derived C4-2B cells were purchased from Urocore (Oklahoma City, OK, USA). LNCaP, C4-2B and PC-3 cells express undetectable levels of PR mRNA and protein. Human cancer associated fibroblasts (hCAFs) were kindly provided by Dr. Simon Hayward (Vanderbilt University). They were derived from human primary cultured cancer associated fibroblasts [2]. Human primary prostate stromal cells, HPS-19I, were generously provided by Dr. David Rowley (Baylor College of Medicine) [4]. Exogenous PRA and PRB were introduced into WPMY-1, hCAF and HPS-19I by lentivirus as described [31]. Protein expression levels as well as cellular localization of PRA and PRB were confirmed (Figure S1). All experiments using hCAFs were within 8–10 cell passages and HPS-19I cells were within 5–6 passages upon received from providers.

### Quantitative Real-Time PCR

Total RNA was extracted using Trizaol (Invitrogen) according to the manufacturer's instructions. Two micrograms of total RNA was subjected to a random-primed reverse transcription using SuperScritpt 2 reverse transcriptase (Invitrogen). Real-time PCR was conducted in triplicates using Applied Biosystem 7900 HT with 5 ng of cDNA, 1 μM of each primer pair and SYBR Green PCR master mix (Roche). The primer sequences were listed in Table S1. Relative mRNA levels were normalized to GAPDH. SiRNA targeting PR was purchased from Thermo Fisher (Cat N. J-003433-08-0005).

### Collection of Conditioned Media

Ninety percent confluent hCAFs, WPMY-1 and HPS-19I stromal cells were washed twice with PBS buffer and replenished with serum free DMEM media in the presence of vehicle, P4 or PR antagonist RU486 for 48 hours. Protein concentrations of conditioned media (CM) were measured by Bicinchoninic Acid assay (Thermo Scientific). The same amount of CM from PR positive and negative cells was used to treat PCa cells for cell migration, invasion and proliferation assays.

### ELISA Assay

SDF-1 and IL-6 concentrations in CM were measured by commercial ELISA kit following manufacturer's protocol (R&D systems, Cat#: DSA00 and D6050).

### Wound Healing Assay

PC-3 cells were grown in medium containing 5% charcoal stripped serum in 6-well plates until 100% confluent. A 20 μl pipette tip was used to scratch to create a wound in the confluent monolayer at the center of dishes. Detached cells were removed by washing with PBS buffer twice. Cells were replenished with 300 ug of CM from hCAFs or 1400 ug of CM from WPMY-1 cells. Cell migration were subsequently captured by an inverted microscope (Axiovert 200 M, Germany) at 0 h and 24 h time points post wound scratch. Experiments were performed in triplicate and repeated three times.

### Cell Migration Assay

PC-3 cells (2.5×10^4^/well) or C4-2B cells (5.0×10^4^/well) were suspended in serum-free DMEM medium and seeded in the BD control chamber without Matrigel (BD Biosciences). Five hundred micrograms of CM from WPMY-1 cells were added to the bottom chamber. After incubation in 37 C with 5% CO2 for 18 hours, non-migratory cells in the upper chamber were gently removed by cotton swabs. Cells that reached the lower chamber were fixed, stained with mounting medium containing DAPI (Vector Laboratories, USA) and photographed under an inverted microscope (Axiovert 200 M, Germany). Cell numbers were counted by the Image J software. Cell migration rate was calibrated to the number of cells incubated with CM from parental WPMY-1 cells as one. Experiments were performed in triplicate and repeated three times.

### Cell Invasion Assay

PC-3 cells (2.5×10^4^/well) or C4-2B cells (1.0×10^5^/well) were suspended in serum-free DMEM medium and seeded in BD Matrigel invasion chamber (BD Biosciences). One hundred micrograms of CM from hCAFs, 500 ug of CM from WPMY-1 cells or 375 ug of CM from HPS-19I cells were added to the bottom chamber. After incubation in 37 C with 5% CO2 for 18 hours, non-invading cells in the upper chamber were gently removed by cotton swabs. Cells that invaded through the Matirgel and reached to the lower chamber were fixed, stained with mounting medium containing DAPI (Vector Laboratories, USA) and photographed by an inverted microscope (Axiovert 200 M, Germany). Invaded cell numbers were counted by the Image J software. Cell invasion rate was calculated by the number of cells invading through Matrigel divided the number of cells migrating through uncoated insert membrane. Experiments were performed in triplicate and repeated three times.

### Chromatin Immunoprecipitation

Chromatin immunoprecipitation assays were performed as previously described [32], [33] with following modifications. Formaldehyde cross-linked chromatin was immunoprecipitated with acetylated Histone 3 antibody (AbCam). Eluted DNA fragments were used as the templates to perform real-time PCR on the ABI PRISM 7900 HT system (Applied Biosystems) using the FastStart Universal SYBR Green Master (Roche). Enrichments of immunoprecipitated DNA fragments were determined by the threshold cycle (Ct) value. Data were calculated as a percentage of input. ChIP data were derived from four independent experiments with samples in triplicate. Data are presented as mean±s.e.m. Primers for SDF-1 promoter are 5′: tggctctcccctctaagc and 3′: ggctgacggagagtgaaagt. Primers for GAPDH promoter are 5′: agtgcctaggctccagatca and 3′: ctcttcccacaaatgctggt.

### Statistical analysis

Data were presented as means ±SEM that were calculated from three or more independent experiments. Statistical significances were calculated by using One-way ANOVA and paired student's t-test using GraphPad Prism (version 4) with the level of significance set at P<0.05 as *, P<0.01 as ** and P<0.001 as ***.

## Results

### PR protein levels are decreased in Prostate Tumors

**Figure 1:**
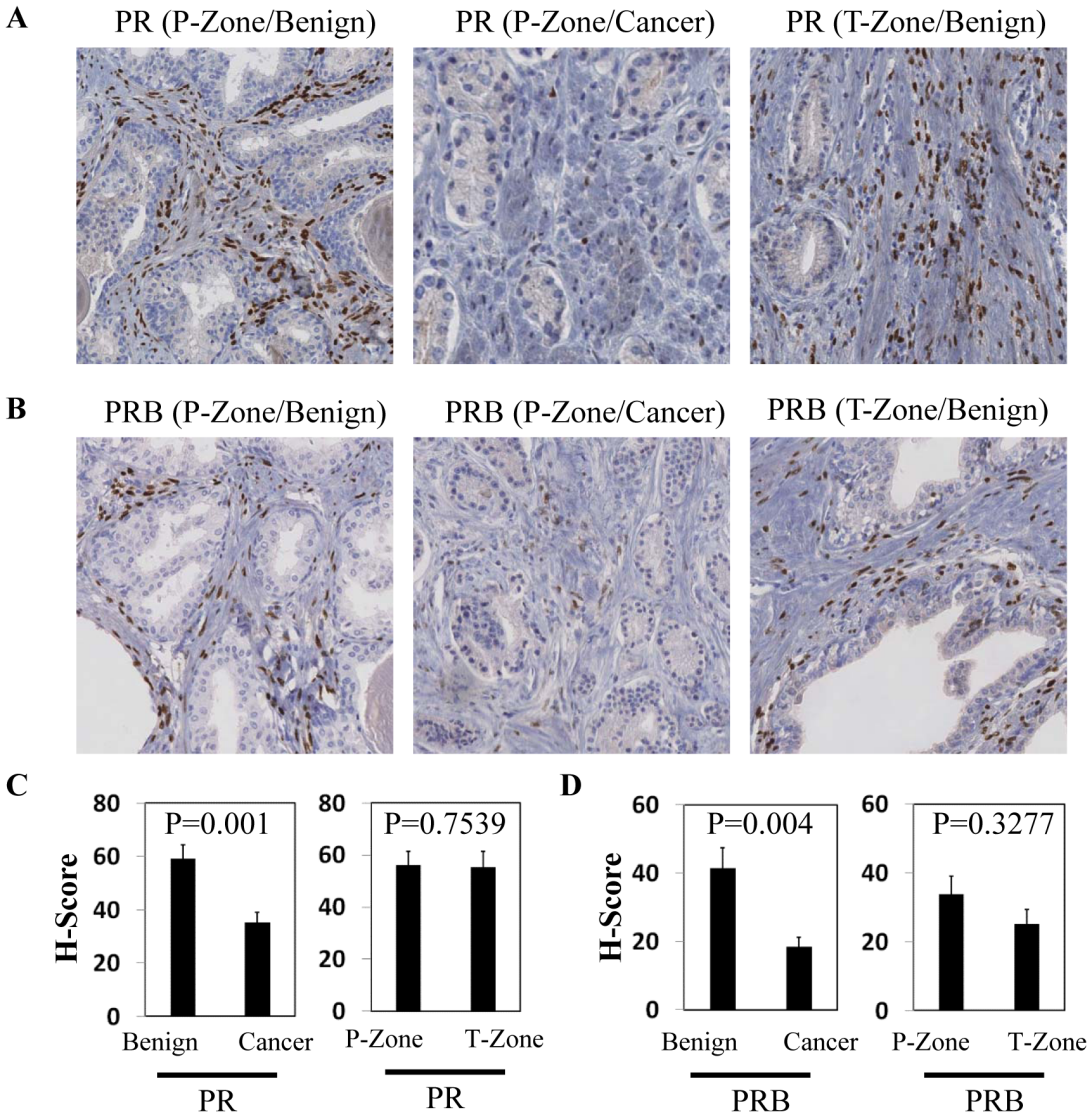
Measurements of PR protein levels in human prostate tumor tissues. Whole mount sections of human prostate biopsies (n = 27) were immunostained with PR antibody (AbCam) or PRB antibody (Cell Signaling). Representative IHC images of PR (**A**) and PRB (**B**) from benign peripheral zones, cancer peripheral zones and transitional zones are shown. IHC staining of PR (**C**) and PRB (**D**) protein levels in paired benign *vs* cancer peripheral zones and in paired benign peripheral *vs* transition zones from 27 whole mount sections of human prostate biopsies were scored by Digital Image Hub (Leica Biosystem). HSCORE indexes were plotted as ±SE. One-way ANOVA and paired student's t-test calculate the level of significance.

We have collected 27 whole mount sections of human prostate tissue biopsies from patients treated with radical prostatectomy (Table 1). The patients are 46 to 88 years in age, with Gleason scores from 6 to 9 and tumor stages ranging from T2A to T3B. Using IHC assays, both total PR and PRB isoforms were detected in the nuclei of a portion of prostate stromal cells (Fig.1A–B). However, HSCORE of PR (combined calculation of intensity and percentile of positive nuclei) was 41% lower (P = 0.001) in cancer associated stroma when compared with paired normal stroma in prostate peripheral zones (Fig.1C). In addition, HSCORE of PRB in cancer associated stroma was about 44% lower than that in normal stroma (P = 0.004) (Fig.1D). Currently, there is no specific antibody to detect PRA isoform. Given the fact that both total PR and PRB decrease in similar degrees, it is likely that the PRA expression levels follow the same trend as PRB. There were no significant differences in either PR or PRB HSCORE between benign peripheral zones and transition zones (Fig.1C–D). There were no statistical differences of total PR or PRB protein levels in association with Gleason score and serum PSA concentrations among these 27 patients. Together, these results indicated that PR levels were decreased in cancer associated stroma.

### Stromal PR inhibits PCa cell migration and invasion

In order to study PR functions at the cellular level, we have applied several human prostatic stromal cell models including hCAFs, WPMY-1 and HPS-19I. These cells express low levels of PR mRNA but undetectable levels of PR protein. We introduced exogenous PRA or PRB isoform in these cells by lentiviral approach as reported [31], which allowed us to study the function of each PR isoform. Although WPMY-1 cells are derived from primary cultured stromal cells from benign prostatic hyperplasia tissues, these cells are immortalized by SV40 large-T antigen. They have similar tumorigenic capacity as hCAFs, when recombined with BPH-1 cells and grafted under mouse renal capsule (unpublished data).

**Figure 2:**
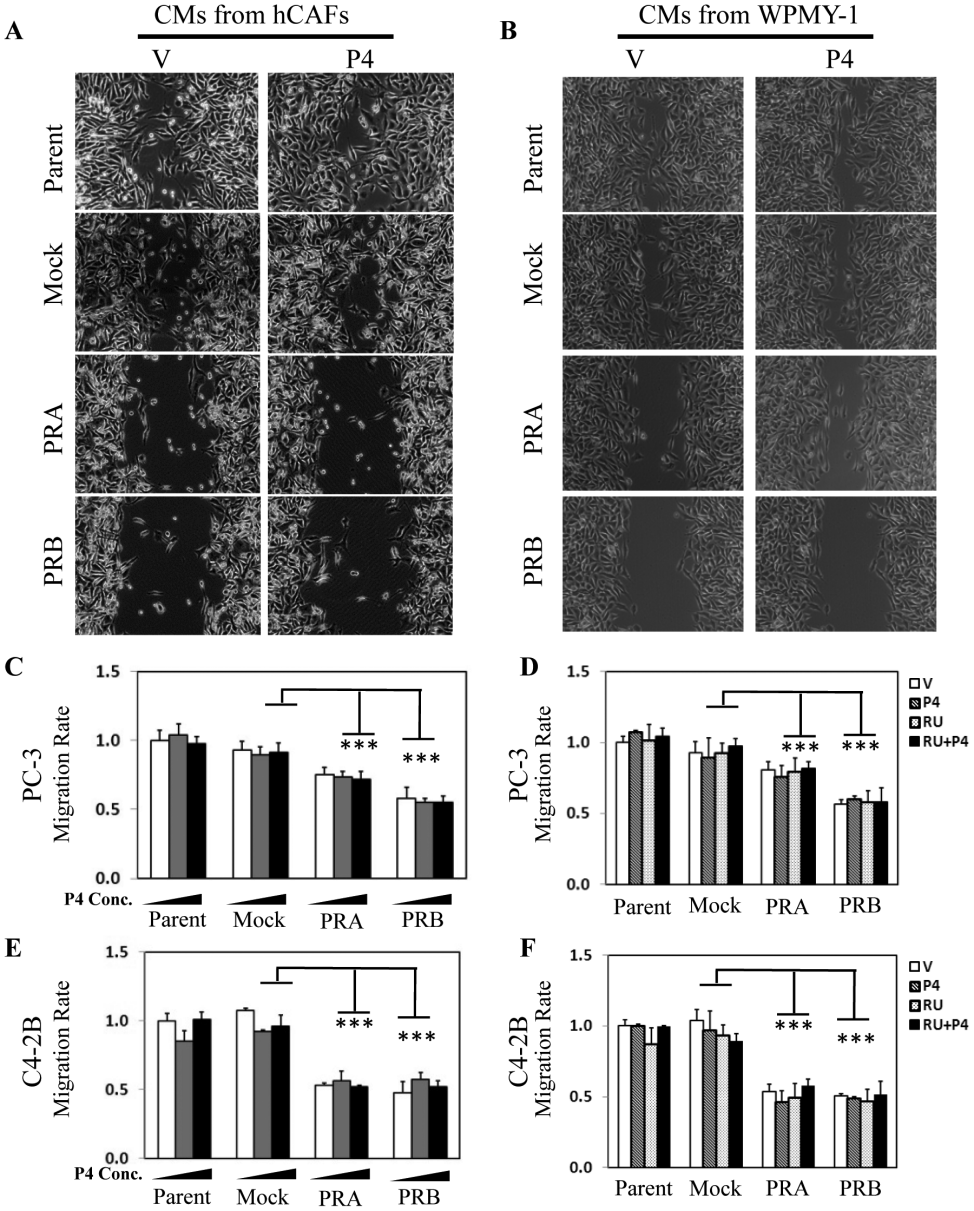
PR negatively regulates prostate cancer migration through a paracrine pathway. Conditioned media (CM) were collected from parental hCAFs, WPMY-1 or their derived cell lines expressing mock, PRA or PRB in the presence of vehicle or 10 nM P4. PC-3 cells were seeded in 6 well plates and incubated with CM from hCAFs (**A**) or from WPMY-1 (**B**) cells for 24 hours in wound healing assays. Representative images after 24 hour CM treatment were captured by an inverted microscope. WPMY-1 and its derived cell lines expressing mock, PRA or PRB were treated with 0, 10 nM and 100 nM of P4 for 24 hours (**C and E**) or with vehicle, 10 nM of P4 and/or 10 uM of RU486 for 24 hours (**D and F**). CM were then collected and incubated with PC-3 cells (**C–D**) and C4-2B (**E–F**) in cell migration assays as described in material and methods section. One-way ANOVA and paired student's t-test calculate the statistical significance set at P<0.05 as * and P<0.001 as ***.

To determine the impact of PR in stromal cells on PCa cell migration, we performed wound healing assays. CM collected from PR-positive and PR-negative hCAFs and WPMY-1 cells in the presence of vehicle or 10 nM P4 were used to incubate with PC-3 cells. Wound healing assays showed that CM from PR positive cells resulted in significantly decreased cell migration (Fig.2A–B). P4 treatment to stromal cells had no impact to the migration rate of PC-3 cells. Fig.2A–B showed representative images from one of three independent experiments. We had also performed cell migration assays to further confirm the ligand-independent action of PR in controlling cancer cell migration. PR positive WPMY-1 cells were treated with increasing doses of P4, and their CM were collected and incubated with PC-3 cells in cell migration assays (Fig.2C). We observed that P4 had no impact on PC-3 cell migration rate, neither did PR antagonist RU486 (Fig.2D). These results were repeatable when we replaced PC-3 cells with the bone metastatic LNCaP-derived C4-2B cells (Fig.2E–F).

**Figure 3:**
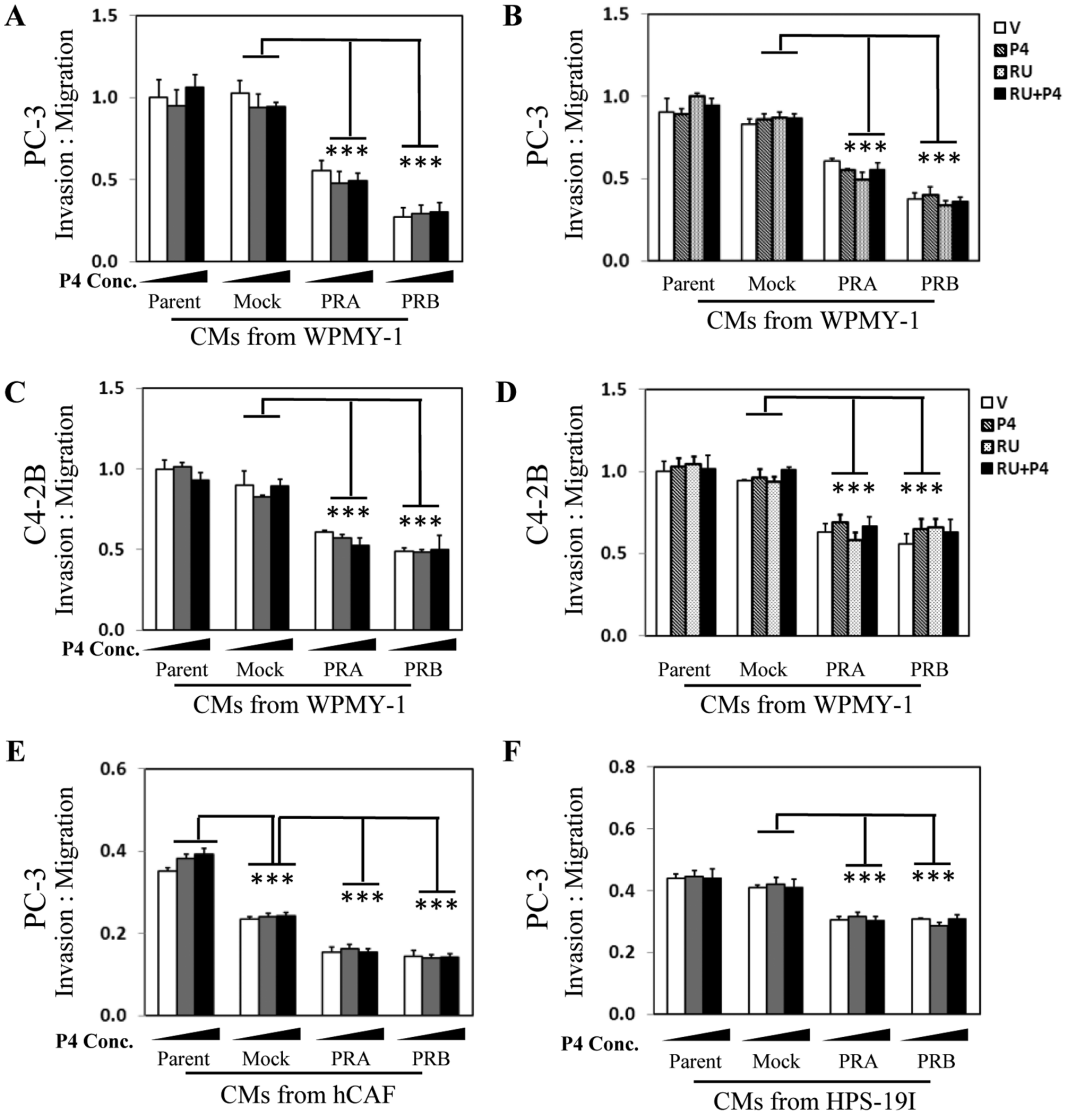
PR negatively regulates prostate cancer invasion through a paracrine pathway. WPMY-1 and its derived cell lines expressing mock, PRA or PRB were treated with 0, 10 nM and 100 nM of P4 for 24 hours (**A and C**) or with vehicle, 10 nM of P4 and/or 10 uM of RU486 for 24 hours (**B and D**). CM were then collected and incubated with PC-3 (**A–B**) and C4-2B (**C–D**) cells in cell invasion assays. hCAFs (**E**), HPS-19I (**F**) cells and their derived cell lines expressing mock, PRA or PRB were treated with 0, 10 nM and 100 nM of P4 for 24 hours. CM were then collected and incubated with PC-3 cells in cell invasion assays. Cell invasion rate was calculated as described in Material and Method section. One-way ANOVA and paired student's t-test calculate statistical significance set at P<0.05 as * and P<0.001 as ***.

To determine the impact of PR in stromal cells on PCa cell invasion, we applied Matrigel invasion assays. CM were collected from PR-positive and PR-negative WPMY-1 cells treated with vehicle, 10 nM or 100 nM of P4 (Fig.3A). We observed that CM collected from PR positive WPMY-1 cells resulted in 50–75% of decrease in PC-3 cell invasion. This PR function was not altered by P4 or by RU486 (Fig.3A–B). In addition, similar results were also observed when C4-2B cell were used in Matrigel invasion assays (Fig.3C–D). Furthermore, we also repeated the experiments with CM collected from two other prostate stromal cells, hCAFs and HPS-19I. We showed that CM from PR positive hCAFs or HPS-19I cells resulted in ∼20–30% of decrease in PC-3 cell invasion, which PR function was also independent to P4 (Fig.3E–F). Together, these results indicated that PR possessed suppressive function to PCa cell migration and invasion in a ligand-independent manner.

To study the role of stromal PR on PCa cell growth *in vitro*, we performed cell proliferation assays (Figure S2). LNCaP cells express a mutant AR that can be stimulated by P4. Treating LNCaP cells directly with P4 in culture medium resulted in 3 fold induction of cell proliferation. CM collected from PR positive hCAFs in the absence of P4 had statistically significant but very mild inhibitory effects on LNCaP cell growth. Similarly, mild suppressive effects were also observed when using CM collected from PR positive WPMY-1 cells in the presence of P4.

### PR represses SDF-1 and IL-6 expression in prostate stromal cells

**Figure 4:**
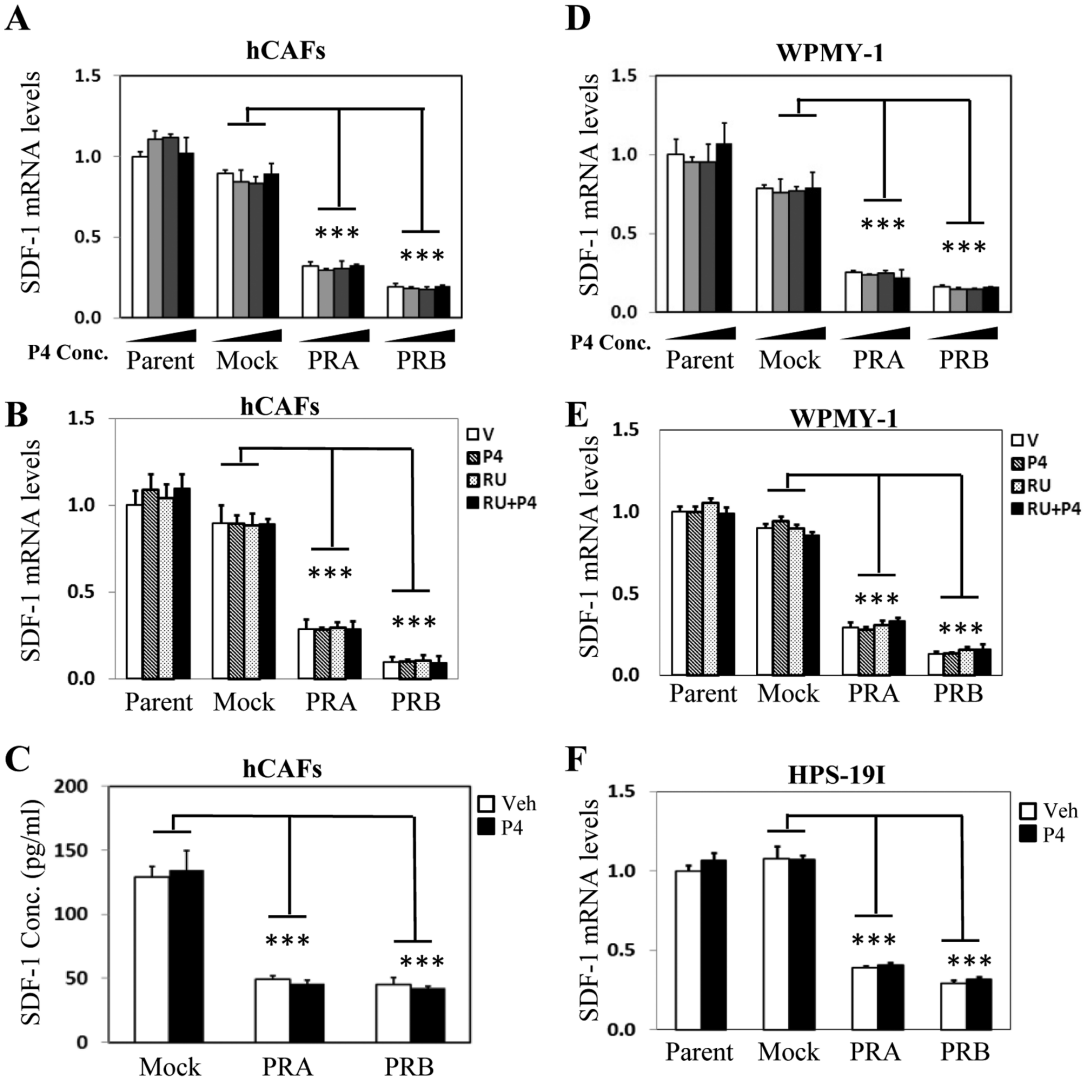
PR supresses SDF-1 expression ligand-independently in prostate stromal cells. hCAFs and their derived cell lines expressing mock, PRA or PRB were treated with vehicle, 1(**A**) or vehicle, 10 nM of P4 and/or 10 uM of RU486 (**B**) for 24 hours. Real-time PCR measured mRNA levels of SDF-1 relative to GAPDH. (**C**) hCAFs and their derived cell lines expressing mock, PRA or PRB were treated with vehicle or 10 nM of P4 for 24 hours. CM were collected and used to measure SDF-1 protein levels by ELISA. WPMY-1 and its derived cell lines expressing mock, PRA or PRB were treated with vehicle, 1 nM, 10 nM and 100 nM of P4 (**D**) or vehicle, 10 nM of P4 and/or 10 uM of RU486 (**E**) for 24 hours. Real-time PCR measured mRNA levels of SDF-1 relative to GAPDH. (**F**) HPS-19I cells and their derived cells expressing mock, PRA or PRB were treated with vehicle or 10 nM of P4 for 24 hours. Real-time PCR measured mRNA levels of SDF-1 relative to GAPDH. One-way ANOVA and followed by student's t-test calculate the significance set at P<0.01 as * and P<0.001 as ***.

**Figure 5:**
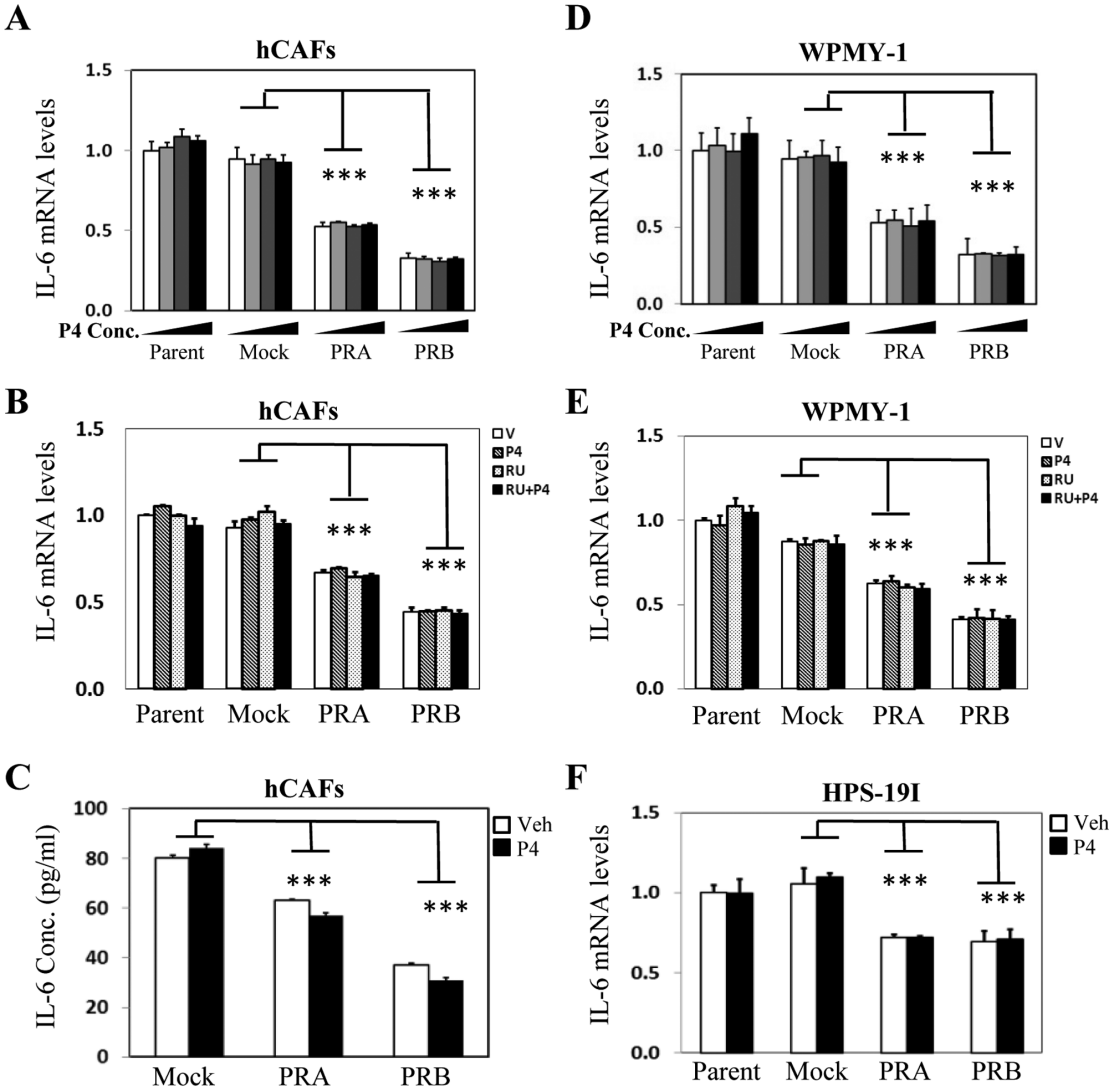
PR supresses IL-6 expression ligand-independently in prostate stromal cells. hCAFs and their derived cells expressing mock, PRA or PRB were treated with vehicle, 1(**A**) or vehicle, 10 nM of P4 and/or 10 uM of RU486 (**B**) for 24 hours. Real-time PCR measured mRNA levels of IL-6 relative to GAPDH. (**C**) hCAFs and their derived cells expressing mock, PRA or PRB were treated with vehicle or 10 nM of P4 for 24 hours. CM were collected and used to measure IL-6 protein levels by ELISA. WPMY-1 cells and their derived cells expressing mock, PRA or PRB were treated with vehicle, 1 nM, 10 nM and 100 nM of P4 (**D**) or vehicle, 10 nM of P4 and/or 10 uM of RU486 (**E**) for 24 hours. Real-time PCR measured mRNA levels of IL-6 relative to GAPDH. (**F**) HPS-19I cells and their derived cells expressing mock, PRA or PRB were treated with vehicle or 10 nM of P4 for 24 hours. Real-time PCR measured mRNA levels of IL-6 relative to GAPDH. One-way ANOVA and student's t-test calculated the significance set with P<0.01 as * and P<0.001 as ***.

Since PR protein level is decreased in cancer associated stroma and CM from PR positive stromal cells inhibit PCa cell invasion and migration *in vitro*, we hypothesize that PR may function to supress secretory factors synthesized by stromal cells and regulate prostate epithelium in a paracrine fashion. In order to test this hypothesis, we re-visited gene microarray data profiling PR regulated genes in prostate stromal cells [31]. By stratifying all of the cytokines and growth factors, we identified SDF-1 and IL-6 as the top ranked genes whose mRNA levels were inhibited by PR. To confirm these findings, we performed real-time PCR and showed that PRA inhibited 70%, while PRB inhibited 80% of SDF-1 mRNA level in hCAFs (Fig. 4A–B). This PR action was ligand independent, as neither P4 nor RU486 had any impact on PR suppression to SDF-1 mRNA levels. Importantly, suppressed SDF-1 mRNA levels by PR resulted in decreased SDF-1 secretion by prostate stromal cells when measured by ELISA (Fig.4C). Furthermore, PR inhibitory action on SDF-1 expression was observed not only in hCAFs, but also in WPMY-1 (Fig.4D-E) and HPS-19I cells (Fig.4F). We also observed similar inhibitory effects of PR to IL-6 mRNA and protein levels in a ligand-independent manner (Fig.5). It is important to be noticed that PR does not present a general suppressive effect to all cytokines or growth factors. Several growth factors including bFGF, HGF, VEGF and KGF are either up-regulated or not altered by PR and/or P4 treatment (Figure S3).

**Figure 6:**
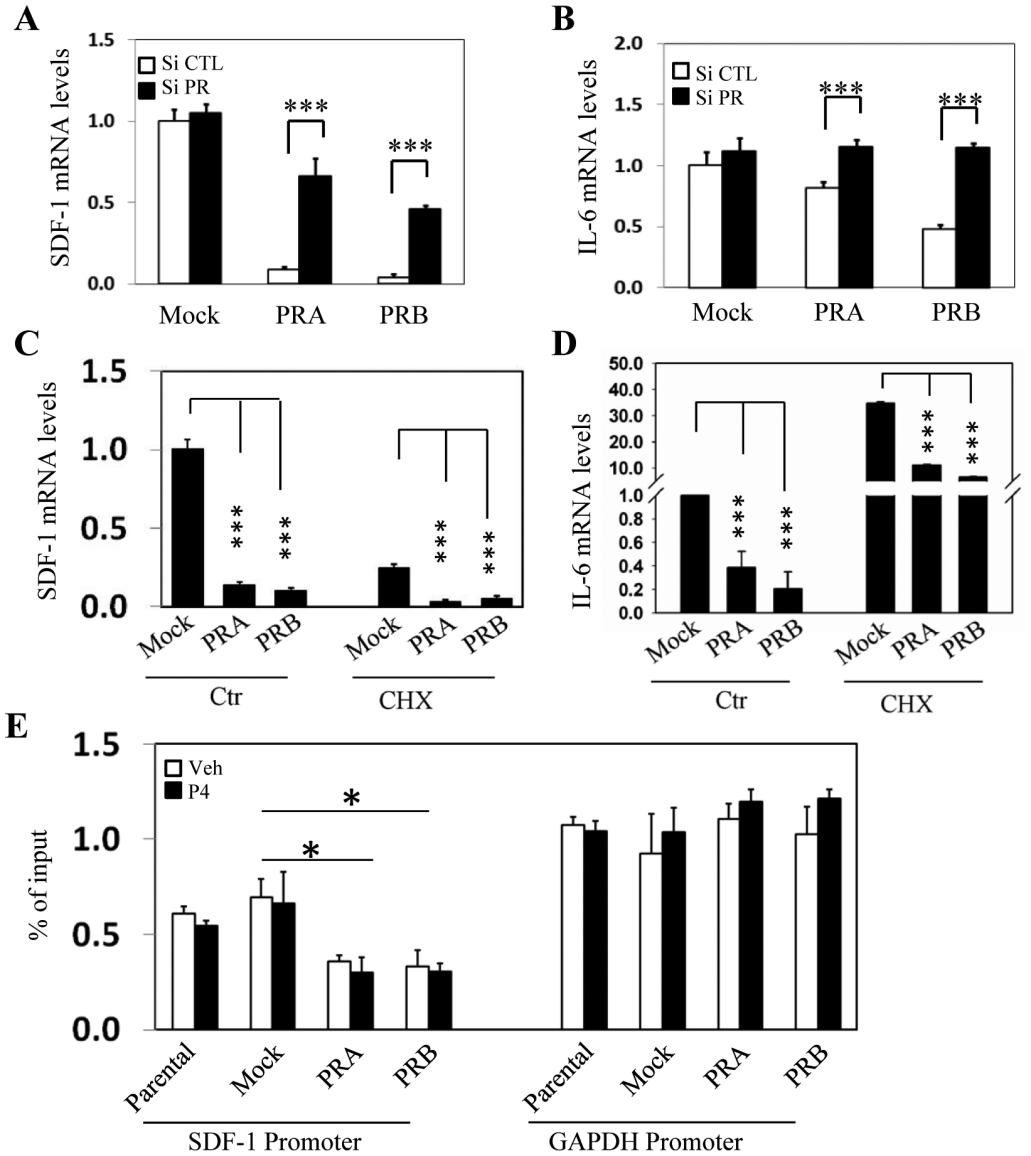
PR represses transcription of SDF-1 and IL-6 genes. WPMY-1 cells expressing mock, PRA or PRB were transiently transfected with control siRNA or siRNA against PR. SDF-1 (**A**) and IL-6 (**B**) mRNA levels relative to GAPDH were measured by real-time PCR. hCAFs expressing mock, PRA or PRB isoform were treated with either control or 20 ug/ml of cycloheximide for 16 hours. Real-time PCR assays measured mRNA levels of SDF-1 (**C**) and IL-6 (**D**) relative to GAPDH. (**E**) WPMY-1 cells and their derived cells expressing mock, PRA or PRB were treated with vehicle or 10 nM of P4 for 24 hours. Chromatin immunoprecipitation assays were performed using acetyl-Histone 3 antibody. Eluted DNA fragments were subjected to measure the enrichment of acetyl-Histone 3 levels in SDF-1 and GAPDH promoter regions. One-way ANOVA and student's t-test calculated the significance set with P<0.01 as * and P<0.001 as ***.

To confirm PR mediated direct inhibition to SDF-1 and IL-6 gene expression, we transiently transfected prostate stromal cells with siRNA against PR (Fig.6A–B). Acute PR knockdown dramatically reversed the inhibitory effects of PR to both SDF-1 and IL-6 mRNA levels. In addition, PR exerted its inhibitory effects directly to SDF-1 and IL-6 gene transcription and did not require other protein synthesis, as PR remained suppressive even in the presence of cycloheximide treatment (Fig.6C–D). We also performed chromatin immunoprecipitation assay to show that histone acetylation levels at the SDF-1 promoter region were dramatically reduced in PR positive stromal cells. These changes were in contrast to histone acetylation status at the GAPDH promoter (Fig.6E). Together, these results support that PR plays a direct role in repressing promoter activities of SDF-1 and IL-6 genes and inhibits their gene transcription.

**Figure 7:**
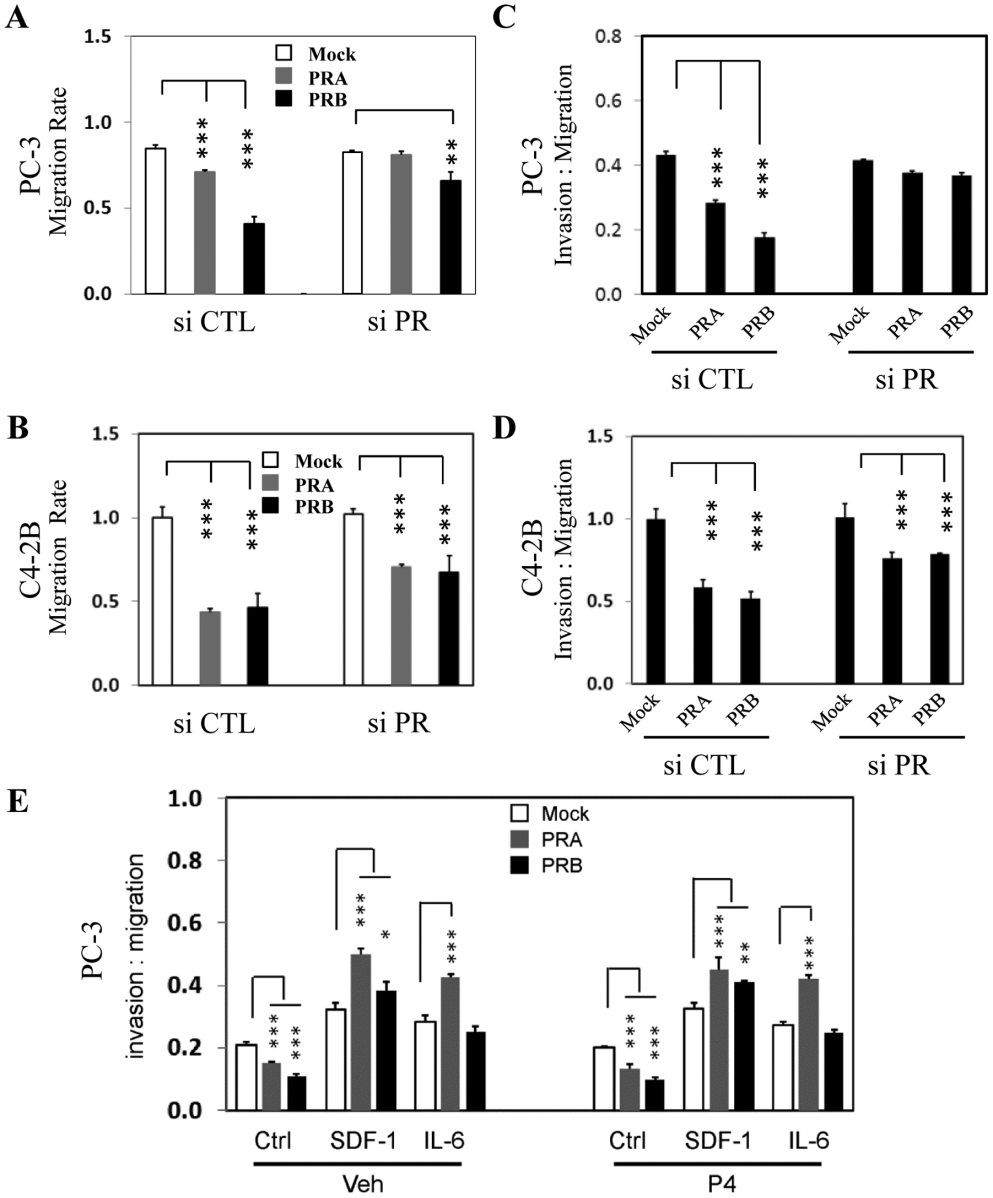
PR inhibitory effects to cancer cell mobility are mediated by SDF-1 and IL-6. WPMY-1 cells expressing mock, PRA or PRB were transiently transfected with control siRNA or siRNA against PR for 24 hours. Cells were washed twice with PBS buffer and replenished with serum free DMEM medium for 48 hours. CM were collected and incubated with PC-3 (**A and C**) or C4-2B (**B and D**) cells for cell migration assays (**A and B**) and Matrigel invasion assays (**C and D**) as described in Material and Method section. (**E**) CM were collected from hCAFs in the presence of vehicle or 10 nM of P4 and then mixed with vehicle, 10 ng/ml of SDF-1 or 10 ng/ml IL-6 and incubated with PC-3 cells in Matrigel invasion assays. One-way ANOVA and paired student's t-test calculate the level of significance set at P<0.05 as * and P<0.001 as ***.

In order to demonstrate that PR-mediated suppression of SDF-1 and IL-6 expression is the major mechanism that reduces PCa cell invasion and migration capacity, we have transiently knocked down PR expression and observed the migration and invasion rates of PC-3 (Fig.7A–C) and C4-2B (Fig.7B–D) cells were recovered. In addition, when recombinant SDF-1 or IL-6 peptide were added to CM, we observed that exogenous SDF-1 or IL-6 abolished PR suppressive effects to PC-3 cell invasion (Fig.7E). Together these results support that PR-mediated suppression to PCa cell mobility is mainly through inhibiting SDF-1 and IL-6 gene expression.

## Discussion

Our studies demonstrate that PR protein levels are decreased in cancer associated stroma and that PR exerts inhibitory impacts to PCa cell mobility through modulating the expression of two important cytokines, SDF-1 and IL-6. These observations suggest that reduced PR expression in cancer associated stroma alters the balanced prostatic microenvironment, which may contribute to PCa cell invasion and metastasis.

The observation that decreased PR expression in cancer associated stroma is similar to what was reported with other steroid receptors such as AR and ER-β [34]–[36], suggesting a general principle that loss of steroid receptors may be required for prostate stroma to be re-activated and to build a supportive microenvironment for PCa. In favoring this hypothesis, stromal AR was shown to suppress PCa cell proliferation and invasion [37]. ER-β agonist enhanced apoptosis of prostate stromal and epithelial cells in aromatase knock-out mice [38]. Although PR and AR share high homology in their DNA binding domains, gene microarray studies have showed different gene profiles regulated by these two receptors [31], [39]. Furthermore, PR is expressed as two major isoforms with identical DNA binding domains. However, they regulate different transcriptome. One explanation could be that PR exerts its transcriptional activity through protein interactions with other transcriptional factors [40], [41].

The mechanism by which PR expression levels are decreased in PCa cells is unknown. Both the intensity of PR staining and the percentile of PR positive nuclei are lower in PCa tissues. Since not all prostate stromal cells express PR, it is unclear whether the PR negative stromal cell population becomes dominant, or whether PR positive cells lose its expression during tumor progression. Our previous work showed that PR positive stromal cells proliferated much slower than PR negative cells [31]. However, it could also be possible that cancer cells might exert paracrine impacts to re-activate prostate stromal cells by supressing their PR expression. These possibilities are not exclusive to each other and may co-exist during cancer development.

Our IHC analyses were applied on whole mount sections of prostate biopsies, rather than tissue microarray (TMA), to measure stromal protein markers. The heterogeneity of prostate stroma requires multiple areas per patient slide to be analyzed by pathologist. This is a crucial standard that cannot be satisfied if using TMA. Due to the small sizes of the tissue cores on TMAs, it is technically difficult to capture representative paired benign and cancer associated stroma and perform pathological comparison analyses.

Ligand-independent actions of PR on gene transcription were reported in several studies [42], [43]. Both liganded and unliganded PR can be located in cell nucleus, but in different sub-nuclear compartments [44], [45]. Posttranslational modifications such as phosphorylation or sumoylation also contribute to PR activation in the absence of progestin [46]. Interestingly, it has been recently reported that AR regulates c-myc expression ligand-independently, which contributes to castration resistant progression of PCa [47]. These findings together suggest that there may be a broader range of genes, whose expression is regulated by steroid receptors independent to ligands. These genes may therefore represent a group of novel molecular targets to block signaling mediated by steroid receptors in PCa.

We identify that SDF-1 and IL-6 are the two important genes, which mediate repressive actions of stromal PR to cancer cells. Decreased PR expression in prostate tumors may result in relatively high levels of SDF-1 and IL-6 secreted by stromal cells, consistent with the reports that SDF-1 and IL-6 levels are elevated in cancer tissue samples [18], [19]. Consistent to the roles of SDF-1 and IL-6 in enhancing tumor cell mobility, we observed that CM from PR positive cells inhibited not only PCa cell migration, but also invasion. In addition, PR has a mild impact on PCa cell growth *in vitro*. LNCaP cell growth is highly dependent upon androgen/AR signaling. Since CM were collected with androgen depleted and serum free medium, cells proliferate at lower rates under such conditions. It is therefore difficult to observe further suppression by CM from PR positive stromal cells. It is also possible that PR may positively regulate other growth hormones, e.g. FGF or HGF, which may neutralize the effects by SDF-1 and IL-6 in cell proliferation.

Our results suggest that PR directly targets SDF-1 and IL-6 gene promoters and inhibit these gene transcriptions. This conclusion is supported by the observation that PR remains suppressive in the presence of the protein synthesis inhibitor cycloheximide. PR specifically reduces the histone acetylation status of SDF-1, but not GAPDH promoter. Furthermore, PR repressive action can be alleviated by transient PR knockdown. Up to date, there was no report on the existence of consensus progesterone response elements in SDF-1 and IL-6 gene promoters. However, it was reported that AP-1 or Sp-1 transcription factor could upregulate SDF-1 transcription [48]. We propose that PR may form a protein complex with AP-1 or Sp-1 to interfere their transcriptional activities. We have reported a similar PR suppressive action on connexin 43 gene transcription [40], [41].

PR positive stromal cells grow much slower than PR negative cells [31], creating an obstacle to study stromal PR functions using PCa xenograft of stromal/epithelial cell recombination. These xenografts require 2–3 weeks to form. Changes in xenograft sizes could be due to altered stromal cell populations by PR or due to suppressed cytokines secretion by PR positive cells. In our *in vitro* cell migration and invasion assays, we treated cells with the same amount of CM rather than co-culture stromal and epithelial cells in order to eliminate the variations of stromal cell numbers. Thus, *in vivo* studies may require tissue recombination technique using urogenital sinus mesenchyme from wild type and PR knockout mice. It was shown that tissue recombination of uterine epithelium with stroma had demonstrated successfully the suppressive effects of stromal PR on DNA synthesis in epithelium [49]. Similar strategy would provide novel insights on the paracrine action of PR in the prostate.

## Supporting Information

Figure S1 (**A**) Exogenous PRA or PRB was introduced into WPMY-1 cells by lentiviral approach. Cellular localization of PR was detected by confocal microscopy as we described [31]. WPMY-1 cells expressing mock, PRA or PRB were transiently transfected with control siRNA or siRNA against PR for 48 hours. PR knockdown efficiency was confirmed by western blotting with PR antibody (**B**) and by real-time PCR (**C**). Note: multiple protein bands were detected by PR antibody due to alternative translation initiation sites, which were characterized previously in Endocrinology 149(11):5872–588.
(TIF)

Figure S2 LNCaP cells were maintained in phenol red free medium with 5% charcoal stripped serum for 48 hours and seeded in 96 well plates (3000 cells/well). Cells were then treated with either vehicle or 10 nM of P4 or incubated with CM collected from hCAFs (upper) or WPMY-1 cells (bottom) as described in Materials and Methods. MTS assays measured cell proliferation rates over 4 days of treatment.
(TIF)

Figure S3 hCAFs expressing mock, PRA or PRB were maintained in phenol red free medium containing 5% charcoal stripped serum for 48 hours. Cells were treated with either vehicle or 10 nM of P4 for 24 hours. Real-time PCR assays measured mRNA levels of bFGF, KGF, HGF and VEGF relative to GAPDH.
(TIF)

Table S1 Primers used in this study.
(TIF)
