# Supplementary figures and images for: Prostate Stromal Cells Express the Progesterone Receptor to Control Cancer Cell Mobility
Source: PLoS One. 2014 Mar 24;9(3):e92714. doi: 10.1371/journal.pone.0092714 (PMC3963951; doi:10.1371/journal.pone.0092714)

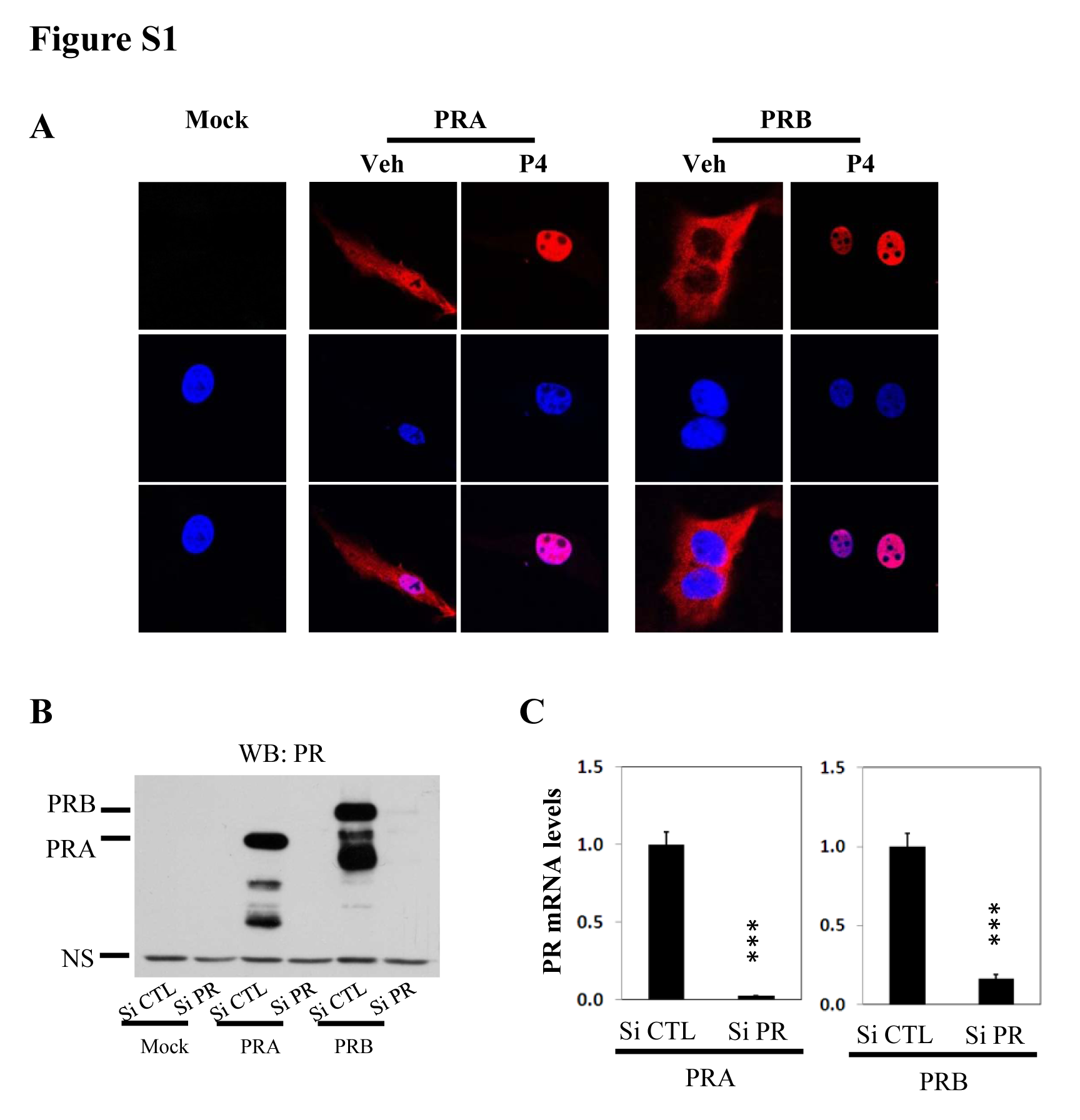

Supplement: Figure S1 — (A) Exogenous PRA or PRB was introduced into WPMY-1 cells by lentiviral approach. Cellular localization of PR was detected by confocal microscopy as we described [31]. WPMY-1 cells expressing mock, PRA or PRB were transiently transfected with control siRNA or siRNA against PR for 48 hours. PR knockdown efficiency was confirmed by western blotting with PR antibody (B) and by real-time PCR (C). Note: multiple protein bands were detected by PR antibody due to alternative translation initiation sites, which were characterized previously in Endocrinology 149(11):5872–588. (TIF) [file pone.0092714.s001.tif]

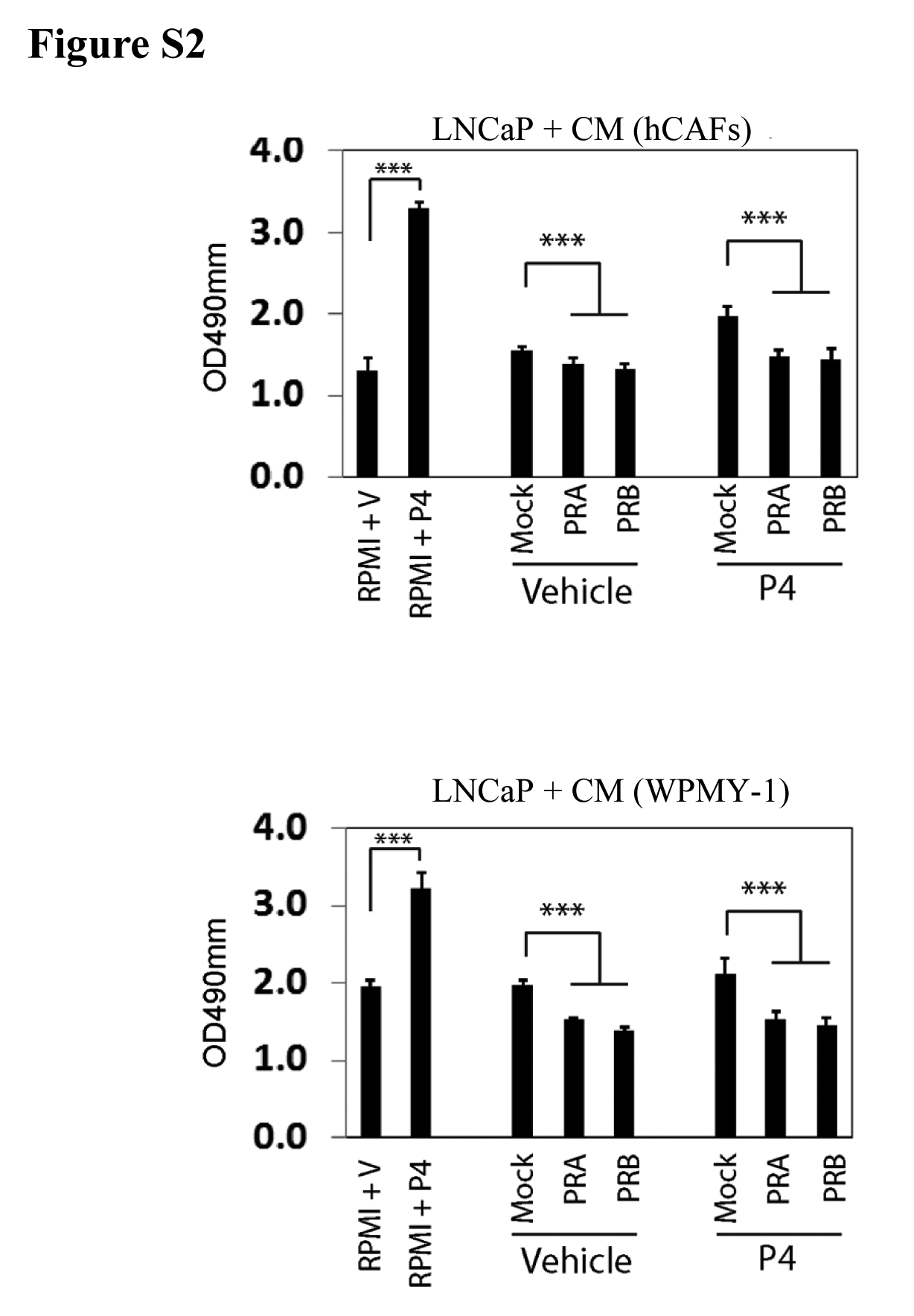

Supplement: Figure S2 — LNCaP cells were maintained in phenol red free medium with 5% charcoal stripped serum for 48 hours and seeded in 96 well plates (3000 cells/well). Cells were then treated with either vehicle or 10 nM of P4 or incubated with CM collected from hCAFs (upper) or WPMY-1 cells (bottom) as described in Materials and Methods. MTS assays measured cell proliferation rates over 4 days of treatment. (TIF) [file pone.0092714.s002.tif]

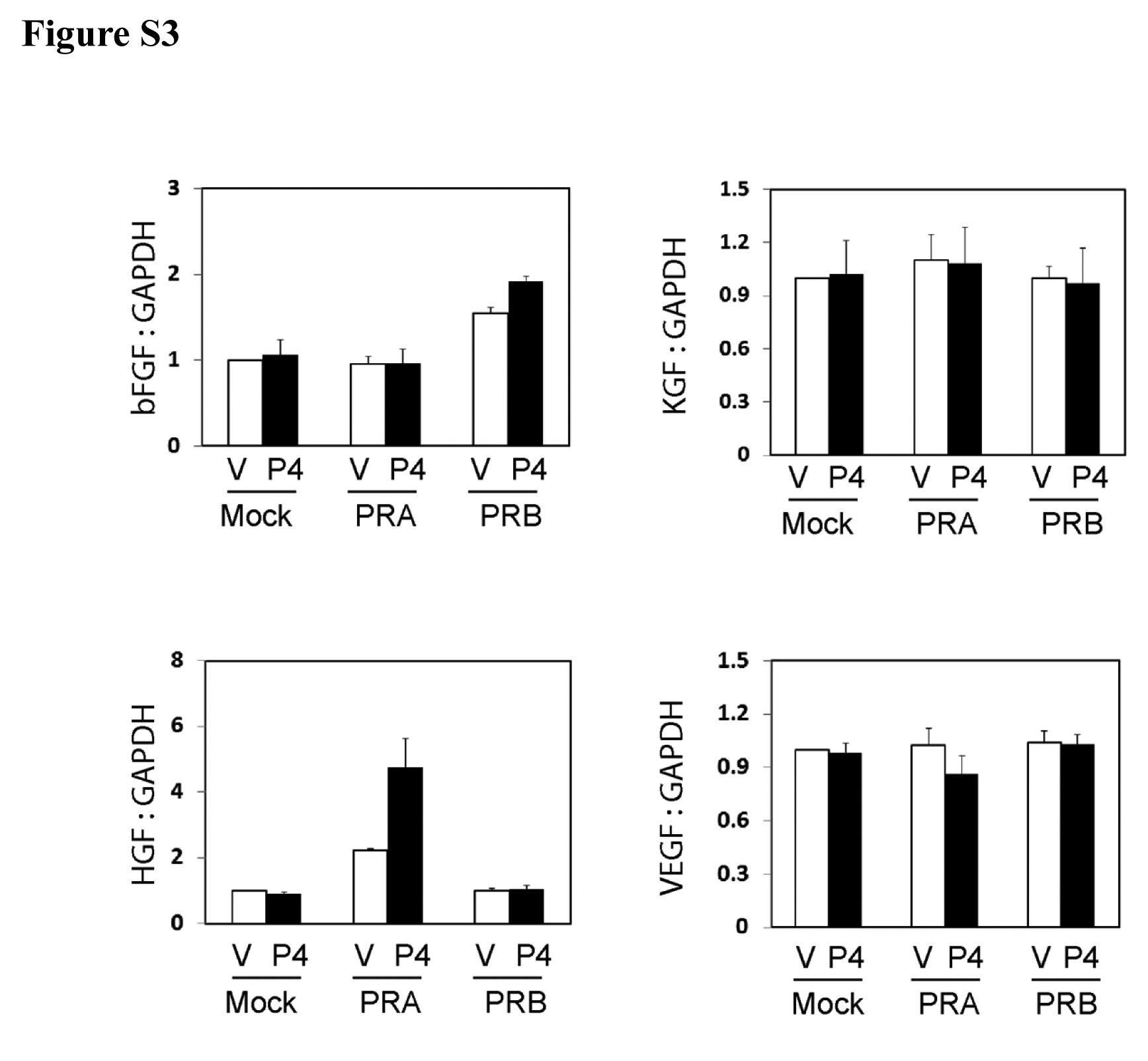

Supplement: Figure S3 — hCAFs expressing mock, PRA or PRB were maintained in phenol red free medium containing 5% charcoal stripped serum for 48 hours. Cells were treated with either vehicle or 10 nM of P4 for 24 hours. Real-time PCR assays measured mRNA levels of bFGF, KGF, HGF and VEGF relative to GAPDH. (TIF) [file pone.0092714.s003.tif]

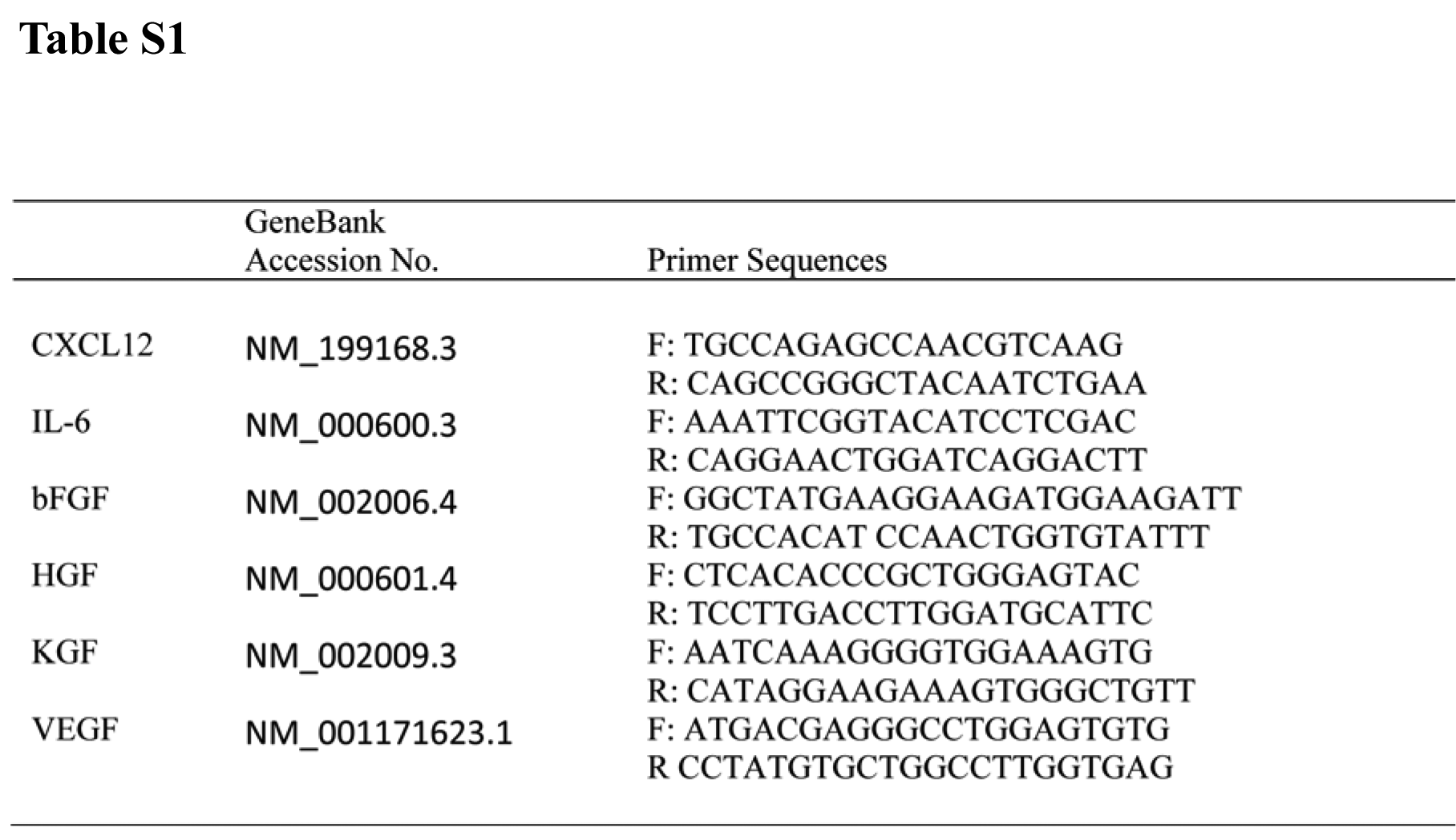

Supplement: Table S1 — Primers used in this study. (TIF) [file pone.0092714.s004.tif]
